# Normative values and integrated score of functional fitness among Chinese community-dwelling older adults in Suzhou

**DOI:** 10.3389/fphys.2022.1063888

**Published:** 2022-12-19

**Authors:** Jing Xu, Ya Chen, Jiaojiao Li, Hui Zhang, Minhao Shi, Hongyan Meng, Li Wang

**Affiliations:** ^1^ The First Affiliated Hospital of Soochow University, Suzhou, China; ^2^ School of Nursing, Suzhou Medical College of Soochow University, Suzhou, China; ^3^ School of Nursing, Vocational Health College, Suzhou, China

**Keywords:** functional fitness, senior fitness test, normative-referenced percentile values, integrated score, older adults

## Abstract

**Objectives:** This study was performed to establish the normative values and integrated score of the functional fitness on the basis of the senior fitness test (SFT) among Chinese community-dwelling older adults in Suzhou.

**Methods:** In this cross-sectional descriptive study, 1,122 community-dwelling older adults aged 60 years old and above were recruited at Suzhou, China, by using a multistage stratified sampling method and accepted the SFT measurements. Sex- and age-specific normative values of each index of the SFT were established by using the percentile method. The SFT integrated score was established using factor analysis according to the data of 70% of the participants (construction group) and verified using the error rate from the data of the remaining 30% of the participants (verification group).

**Results:** Normative-referenced percentile values at the 5th, 10th, 25th, 35th, 50th, 65th, 75th, 90th, and 95th percentiles for each index of SFT were established for the men and women among the different age groups. Five indices of the SFT, namely, 2-min step test, 30-s arm curl, 30-s chair stand, chair sit-and-reach, and 8-ft up-and-go (TUGT), gradually declined with age in both sexes (*p* < .05). The SFT integrated score was calculated as follows: F = 3.8 × 2-min step test + 3.8 × 30-s arm curl + 3.8 × 30-s chair stand + 2.2 × back starch + 2.6 × chair sit-and-reach + 4 × TUGT − .04 × BMI. The formula was verified using the error rate. The error rates of the verification group compared with the construction group in each grade score of SFT were lower than 5%.

**Conclusion:** Based on the data from the community-dwelling older adults in Suzhou, China, the functional fitness normative values for each index of the SFT and the integrated score of SFT were established. The SFT integrated score formula was verified to be reasonable and effective.

## 1 Introduction

The aging of population has become a significant social problem in China. According to the National Bureau of Statistics, the number of people aged 60 years and over was 264.02 million by the end of 2020, and these people accounted for 18.7% of China’s total population. Among this older population, the number of people aged 65 years or above was 190.64 million (i.e., 13.5% of China’s total population) (Ning, 2021). In China, Suzhou is one of the earliest areas to enter aging and also one of the most serious aging areas (Mao et al., 2014). Data from the seventh population census in 2020 show that the population aged 65 years and above accounted for 12.44%, with an increase of 3.94 percentage points compared with the sixth population census in 2010. The elderly population in Suzhou has been predicted to exceed 2.5 million by 2030, and the proportion will rise to 37.4% (Zhu et al., 2022). Aging results in the decline of physical function. This characteristic seriously affects the daily activity ability, causing health events, such as falls, fractures, and even the high risk of disability and death, in older adults.

Functional fitness comprehensively and effectively reflects the physical function of older adults (Osness, 1990). The major components of functional fitness include aerobic fitness, muscle strength, flexibility, and motor agility/dynamic balance (Osness, 1990). Some of the common tests currently used in evaluating the functional fitness of older adults are the Short Physical Performance Battery, five-repetition sit-to-stand test, and walking speed test. However, these assessments are not sensitive enough to distinguish small changes in physical function (Li et al., 2013). In addition, all these tests have a “ceiling” or “floor” effect, so they could not accurately reflect the functional fitness level of the elderly or is only applicable to people with specific physical conditions (Li et al., 2013). The American Alliance for Health, Physical Education, Recreation, and Dance has designed a series of tests to assess the functional fitness in the elderly. However, the lower limb strength test is missing, and the series has the “ceiling” effect in which some indices are difficult for older adults to complete (Yaguchi and Furutani, 1998).

The senior fitness test (SFT), which is a battery test containing all dimensions of functional fitness, has been widely used around the world for evaluating functional fitness (Rikli and Jones, 2013). This assessment includes simple and practical tests with moderate intensity, which is suitable for a wide range of older adults. After the earliest study of the normative values of the SFT in older adults in the United States (Rikli and Jones 1999b), the SFT normative values has been established in populations in several other countries, such as Germany (Albrecht et al., 2021), Chile (only in females) (Valdés-Badilla et al., 2018), Spain (Gusi et al., 2012), Portugal (Marques et al., 2014), and Poland (Ignasiak et al., 2020). In addition, the SFT integrated score has been calculated and used to assess the physical function in Portugal and Finland (Sardinha et al., 2016; Jantunen et al., 2017).

However, the application of the SFT in China is currently limited to primarily being an outcome parameter in evaluating changes in the physical function before and after intervention. To the best of our knowledge, five studies have investigated the normative values of the SFT or part of the indices of the test in Chinese population (Chen et al., 2009; Wang, 2015; Chung et al., 2016; Lin, 2020; Zhao et al., 2021). Among these reports, three earlier ones had participants from Hong Kong (HK) (Chung et al., 2016), Taiwan (Chen et al., 2009) and Anhui (Wang, 2015), while the two more recent ones had participants from Nanjing (Zhao et al., 2021) and Lanzhou (Lin, 2020). Only one study from Lanzhou has constructed comprehensive evaluation criteria, but the functional fitness measurement parameters were not identical with those of the SFT (Lin, 2020). Lanzhou is located in northwest China, which is a transitional area from the Qinghai Tibet Plateau to the Loess Plateau. Most areas in the territory are at an altitude of 1,500–2,500 m, which is quite different from the plain area. The study in Taiwan contained only four indices of the SFT (Chen et al., 2009), and the one in Anhui contained more than 20 indices and covered only the older adults over the age of 70 years (Wang, 2015).

The purpose of this study was to establish the SFT normative values and integrated score by investigating the community-dwelling older adults in Suzhou, China. With the laboratory and institution located at Suzhou, we have certain advantages in organizing the measurement and handling potential problems. Through this study, we aim to provide complementary data to the existing SFT normative values and fill a gap in the construction of the SFT integrated score. Thus, we anticipate to provide a reference standard for evaluating the overall physical function.

## 2 Methods

### 2.1 Sample size estimate

For the sample size calculation, the standard deviation (σ) and permissible error (E) (≤5%) of three indices, namely, the 2-min step test for aerobic endurance, 30-s arm curl, and 30-s chair stand, which constituted the two main aspects of functional fitness (aerobic endurance and muscle strength), were obtained from the pretest from out lab (unpublished data). The corresponding σ and E values were 17.58 and 2.15 in the 2-min step test, 5.88 and .45 in the 30-s arm curl, and 3.30 and .36 in the 30-s chair stand. These data were substituted into the following formula.

$$n=\frac{{\left(Z_{\alpha /2}\right)}^{2}\sigma^{2}}{E^{2}}$$

Zα/2: the critical value for a 95% confidence interval, which equals to 1.96; σ: standard deviation; E: permissible error.

The corresponding calculated sample sizes for the three indices were 257, 668, and 321. The maximum size of 668 was adopted. Considering a 10% dropout rate (more than three uncompleted SFT indices were regarded as shedding samples), the final sample size was set to 742.

### 2.2 Participants

In this cross-sectional descriptive study, participants who met the inclusion criteria were recruited and accepted the SFT measurements. Community older adults aged 60 years and above were recruited from Suzhou in China from October 2020 to November 2021 by using a multistage stratified sampling method. In the first stage, the six municipal districts of Suzhou (namely Wuzhong, Gusu, Xiangcheng, Wujiang, Huqiu and Industrial Park) were all selected. In the second stage, 1–3 streets (sub-districts) were selected in each municipal district with convenience sampling method and then 1–2 Community Healthcare Centers were selected in each of the street with the same method. Finally, the older adults were recruited from the selected Community Healthcare Centers using convenience sampling. The elderly who came to the Community Healthcare Centers for physical examination were asked face to face about their willingness to accept the fitness test and participate in the study. Meanwhile flyers were posted in Community Healthcare Centers to improve the awareness and understanding of the study in the community residents.

A total of 1,122 older adults with an age range of 60–93 years were finally included. The inclusion criteria for the older adults were as follows: aged ≥60 years; community residing; functionally independent and could walk independently without assistive device; conscious and could communicate; and willing to sign an informed consent form. The exclusion criteria were older adults with contraindications to exercise. The drop-out criteria were any older adults who could not complete at least three tests within the SFT.

The study was conducted in accordance with the guidelines of the Declaration of Helsinki and was approved by the Ethics Committee of Soochow University (SUDA20210630H02). All participants were fully informed of the research purpose, content, and risks before signing a consent form.

### 2.3 Random method

Data from all participants were included to establish the SFT normative values for different sexes and ages. Meanwhile, the participants were randomly divided into the construction and verification groups at a ratio of 7:3, which was used in a previous study (Yoon et al., 2021). The construction group was used to develop the SFT integrated score, and the verification group was used for the validity test. The random method was as follows. The participants were numbered according to the order of inclusion, and random numbers were generated by IBM SPSS 23.0 software. Thus, each participant corresponded to a random number. Then, the random numbers were arranged from small to large, and the participants were divided into the construction (70%) and verification (30%) groups.

### 2.4 Measures

The participants completed self-designed questionnaires on sociodemographic data, health-related data, and lifestyle information (Table 1) during a face-to-face interview. Among the questions investigated, regular exercise referred to the performance of exercise of no less than thrice a week, with each instance lasting for no less than 20 min (Cho and Kim, 2020). Physical activity referred to the occupational, leisure, household, and other daily activities, such as laundry, cooking, sweeping, mopping, and cleaning the yard (Caspersen, 1989). The participants chose the appropriate options according to their actual situation.

**TABLE 1:** Characteristics of participants, *n* (%) or mean ± SD.

Variables	Total	Construction group	Verification group	*χ* ^*2*^/*t*	*p*
*N*	1,122	785	337		
Sex					
Men	458 (40.8)	321 (40.9)	137 (40.7)	.006^a^	.941
Women	664 (59.2)	464 (59.1)	200 (59.3)		
Age (years)				1.589^a^	.811
60–64	306 (27.2)	214 (27.3)	92 (27.3)		
65–69	334 (29.8)	236 (30.1)	98 (29.1)		
70–74	233 (20.8)	168 (21.4)	65 (19.3)		
75–79	159 (14.2)	107 (13.6)	52 (15.4)		
≥80	90 (8.0)	60 (7.6)	30 (8.9)		
Height (m)	1.61 ± .08	1.61 ± .08	1.61 ± .08	−.318^b^	.751
Weight (kg)	62.22 ± 10.30	62.00 ± 10.26	62.73 ± 10.39	−1.067^b^	.286
Educational level				1.396^a^	.707
Illiteracy	147 (13.2)	105 (13.4)	42 (12.7)		
Primary school	275 (24.6)	196 (25.0)	79 (23.8)		
Middle school	343 (30.7)	245 (31.2)	98 (29.5)		
High school and above	352 (31.5)	239 (30.4)	113 (34.0)		
Marriage status				.321^a^	.571
Married	945 (84.2)	661 (84.2)	284 (85.5)		
Others	172 (15.3)	124 (15.8)	48 (14.5)		
Economic sources				1.386^a^	.500
Labor income	125 (11.1)	83 (10.6)	42 (12.7)		
Retirement pension	848 (75.6)	597 (76.1)	251 (75.6)		
Others	144 (12.8)	105 (13.4)	39 (11.7)		
Medical payment method				5.303^a^	.071
Self-paid	27 (2.4)	16 (2.0)	11 (3.3)		
Medical insurance	971 (86.9)	676 (86.1)	295 (88.9)		
Others	119 (10.7)	93 (11.8)	26 (7.8)		
Occupation				.439^a^	.507
On job	260 (23.2)	187 (23.8)	73 (21.7)		
Retirement	857 (76.4)	598 (76.2)	259 (76.9)		
Regular exercise				.059^a^	.808
No	387 (34.7)	270 (34.5)	117 (35.2)		
Yes	728 (65.3)	513 (65.5)	215 (64.8)		
Physical activity				.199^a^	.655
No	158 (15.0)	113 (15.3)	45 (14.2)		
Yes	896 (85.0)	625 (84.7)	271 (85.8)		
Complication				.025^a^	.875
No	166 (14.8)	117 (14.9)	49 (14.5)		
Yes	956 (85.2)	668 (85.1)	288 (85.5)		

^a^ Chi-squared test.

^b^ Independent samples *t* test.

Functional fitness was assessed using the SFT, which was designed and validated by Rikli and Jones (1999). SFT consists of six compulsory indices of 2-min step test (aerobic endurance), 30-s arm curl and 30-s chair stand (upper and lower extremity muscle strength), back scratch and chair sit-and-reach test (upper and lower body flexibility), 8-ft up-and-go (TUGT, for agility and dynamic balance), and one recommended index of body mass index (BMI). Each index was tested strictly in accordance with the Senior Fitness Test Manual (Rikli and Jones, 1999a). The reliability range of the SFT indices was .80–.97, and the validity range was .79–.9 (Rikli and Jones, 2013). The values of the minimal detectable changes in community-dwelling older adults are currently not available. However, in older adults with cognitive impairment, minimal values for detectable change at the 90% confidence intervals were calculated to be as follows: 30-s chair stand, 2.0 reps.; 30-s arm curl, 2.3 reps.; chair sit-and-reach, 6.0 cm; back scratch, 4.6 cm; TUGT; 1.4 s; and 6MWT, 37.1 m (Hesseberg et al., 2015).

All tests were conducted by the members of the research team who accepted a unified training. The training covered questionnaire filling, instrument usage, and standardized test of each index to ensure their proficiency in the use of instruments and accuracy of measurement.

### 2.5 Statistical analysis

Data were analyzed using SPSS 23.0 (IBM, Armonk, NY, United States). All continuous data are presented as the mean ± standard deviation (SD) if normally distributed; otherwise, the median was used. All categorical data are presented as frequencies and percentages (%). To compare the difference between the construction and verification groups, chi-squared test was used for the categorical variables and independent samples *t*-test for the quantitative variables.

Participants were divided into five age groups (AGs): AG1, 60–64 years old; AG2, 65–69 years old; AG3, 70–74 years old; AG4, 75–79 years old; and AG5, ≥80 years old. Two-way ANOVA was conducted to explore the interaction between AGs and sex in each functional fitness index. One-way multivariate ANOVA was used to examine the sex differences of each index in different AGs and the age differences in women and men. Pairwise comparison was then performed to reveal the differences between any two adjacent AGs (i.e., AG1 and AG2, AG2 and AG3, AG3 and AG4, and AG4 and AG5). The effect size (ES) was calculated using Hedges’g, where g was the mean difference of two comparison groups divided by the weighted and pooled standard deviation (Cohen, 2013). The following cut-off values for the ES were considered: ES ≤ .20, low; ES > .20 and <.80, moderate; ES ≥ .80, high (Thomas et al., 2015). The decline rate was calculated as (AG5 − AG1)/AG1 × 100.

The normative value was constructed for seven indices of SFT by using the percentile method and was calculated for AG1–5 in women and men. The SFT integrated score was obtained based on the weight distribution deduced from the factor analysis method and divided into five grade scores of 1–5. The error rates between the construction and verification groups (differences in the percentages of participants for each SFT grade score between the construction and verification groups) were calculated, and the smaller the difference was, the higher was the consistency. An error rate not higher than 5% was up to standard (Tu et al., 2021), and *p* < .05 was considered statistically significant.

## 3 Results

### 3.1 Characteristics of participants

**FIGURE 1:**
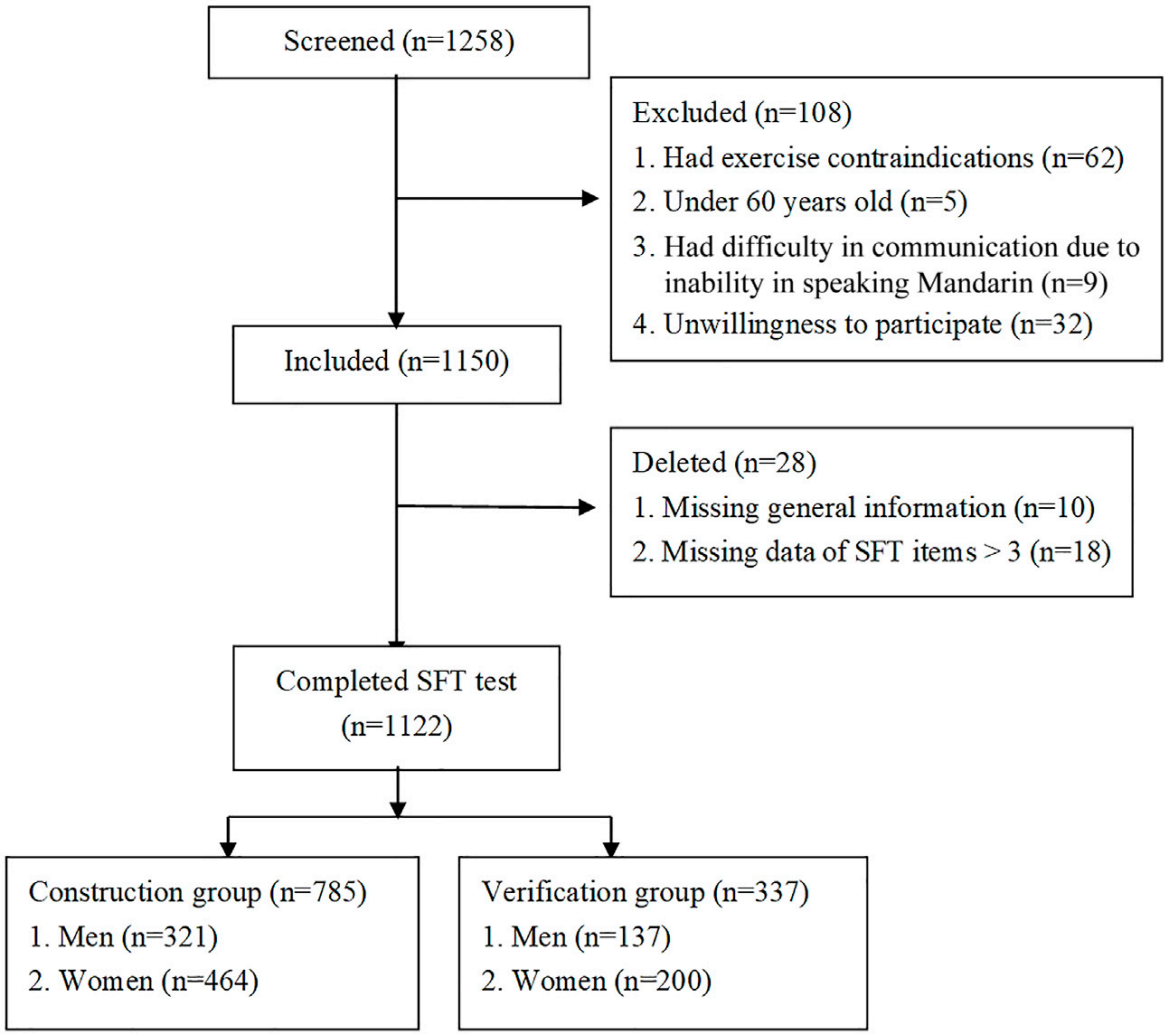
Flow chart of participants.

A total of 1,258 older adults above 60 years old were screened, but 108 were excluded. Among the remaining 1,150 older adults, data of 28 older adults (2.4%) were deleted due to incompleteness. Finally, 1,122 (97.6%) older adults (458 men and 664 women, aged 60–92 years old) were included in this study, among whom 785 were assigned into the construction group and 337 into the verification group (Figure 1).

The proportion of male and female older adults in different AGs in our study and in Suzhou city (obtained and calculated from the data of the seventh population census in 2020 in the webpage of https://www.hongheiku.com/shijirenkou/557.html) is presented in Supplementary Table S1. The proportions of each AG in our study were similar to the ones in the city except a smaller percentage in the AG of ≥80 years old. The male and female older adults account for 40.8% and 59.2% in our study, and 47.8% and 52.2% in the city.

The general characteristics of the 1,122 participants and the participants in construction and verification groups are listed in Table 1. AG1–3 consisted ∼80% of the total sample population. Nearly one-third (31.5%) of the participants had an educational level of high school and above. The majority of participants were retired and had regular exercise or physical activities. No significant differences were found in the general characteristics between the construction and verification groups (*p* > .05) (Table 1).

### 3.2 Sex and age effects on the functional fitness index

The mean values of the seven indices within the SFT in each AG for men and women are presented in Table 2. Comparison of the ES values of the sex and AGs are listed in Table 3.

**TABLE 2:** Results of each SFT index among five different age groups in men and women, mean ± SD.

Items	Age group (years old)					*F1*	*F2*
	AG1 (60–64)	AG2 (65–69)	AG3 (70–74)	AG4 (75–79)	AG5 (≥80)		
2-min step test (reps.)							
Men	92.82 ± 15.86	89.28 ± 15.86	86.56 ± 16.73^a^	84.04 ± 14.96^a^	76.38 ± 16.17^abc^	10.519**	.148
Women	94.72 ± 15.21	91.29 ± 15.28	84.79 ± 18.26^ab^	83.40 ± 16.64^ab^	76.98 ± 18.80^ab^	17.216**	
30-s arm curl (reps.)							
Men	19.45 ± 3.37	18.24 ± 3.41	17.42 ± 3.17^a^	16.45 ± 3.19^ab^	15.31 ± 3.43^abc^	18.140**	.200
Women	19.14 ± 2.80	18.45 ± 3.13	17.36 ± 3.65^ab^	16.17 ± 3.49^ab^	15.26 ± 3.81^abc^	21.952**	
30-s chair stand (reps.)							
Men	17.42 ± 3.36	16.31 ± 3.52	15.20 ± 3.62^a^	14.77 ± 3.07^ab^	13.40 ± 3.88^abc^	15.256**	2.091
Women	17.68 ± 3.29	15.75 ± 3.10^a^	15.00 ± 3.65^a^	13.70 ± 3.00^ab^	13.31 ± 3.69^abc^	31.378**	
Back scratch (cm)							
Men	−9.58 ± 10.03	−11.35 ± 11.86	−12.44 ± 11.19	−10.12 ± 11.54	−12.32 ± 12.87	1.156	63.428**
Women	−2.47 ± 9.41^#^	−4.11 ± 9.82^#^	−5.09 ± 9.73^#^	−7.40 ± 10.93^a^	−8.30 ± 12.16^a^	5.362**	
Chair sit-and-reach (cm)							
Men	−1.14 ± 10.71	−2.02 ± 10.69	−3.98 ± 11.91	−3.42 ± 9.57	−6.65 ± 11.12^a^	2.692*	65.841**
Women	4.30 ± 9.28^#^	3.76 ± 7.59^#^	2.03 ± 9.24^#^	−.24 ± 9.00^ab#^	−.61 ± 9.23^ab#^	6.366**	
TUGT (s)							
Men	5.58 ± .91	5.95 ± .87^a^	6.41 ± 1.27^ab^	6.80 ± 1.57^ab^	8.12 ± 1.97^abcd^	40.793**	3.367
Women	5.45 ± .72	5.94 ± .89^a^	6.49 ± 1.04^ab^	6.81 ± 1.35^ab^	7.48 ± 1.78^abc^	53.620**	
BMI (kg/m^2^)							
Men	24.60 ± 2.80	24.21 ± 2.95	24.75 ± 2.94	24.48 ± 2.98	24.12 ± 2.76	.689	23.406**
Women	23.57 ± 2.90^#^	24.04 ± 3.09	23.61 ± 2.85^#^	23.25 ± 3.44^#^	22.83 ± 2.80^#^	2.095	

*p < .05, **p< .001, ^a,b,c,d:^ *p* < .05 vs*.* AG1, 2, 3 and 4 respectively, ^#^ *p* < .05 vs*.* men.

*F1*: statistic for age group comparison; *F2*: statistic for sex comparison. *n* = 118, 119, 95, 78, and 48 for men in AG1-5 respectively and *n* = 188, 215, 138, 81 and 42 for women in AG1-5 respectively.

**TABLE 3:** Effect size (ES) and decline rate of each SFT index.

Items	Age group (years old)					%Decline
	AG1 (60–64)	AG2 (65–69)	AG3 (70–74)	AG4 (75–79)	AG5 (≥80)	
2-min step test (reps.)						
Men		.22	.17	.16	.49	17.71
Women		.23	.39*	.08	.37	18.73
Men vs*.* Women	.12	.13	.10	.04	.03	
30-s arm curl (reps.)						
Men		.36	.25	.30	.35	21.29
Women		.23	.33*	.33	.25	20.27
Men vs*.* Women	.10	.07	.02	.08	.01	
30-s chair stand (reps.)						
Men		.32	.31	.13	.40	23.08
Women		.60**	.23	.38	.12	24.72
Men vs*.* Women	.08	.17	.06	.35	.02	
Back scratch (cm)						
Men		.16	.09	.20	.18	28.60
Women		.17	.10	.23	.08	236.03
Men vs*.* Women	.74**	.68**	.71**	.24	.32	
Chair sit-and-reach (cm)						
Men		.08	.17	.05	.32	483.33
Women		.06	.21	.20	.04	114.19
Men vs*.* Women	.55**	.65**	.58**	.34*	.58*	
TUGT (s)						
Men		.41*	.43*	.28	.76*	45.52
Women		.60**	.58**	.27	.44	37.25
Men vs*.* Women	.16	.01	.07	.01	.34	
BMI (kg/m^2^)						
Men		.14	.18	.09	.12	1.95
Women		.16	.14	.12	.13	3.14
Men vs*.* Women	.36*	.06	.39*	.38*	.46*	

*p < .05, **p< .001.

The values in the lines of “Men” and “Women” means the effect size of the current age group compared with the age group prior. The values in the line of “Men vs*.* Women” means the effect size of women compared with men.

Five indices of the SFT, namely, the 2-min step reps., 30-s arm curl reps., 30-s chair stand reps., chair sit-and-reach, and TUGT result, gradually declined with age in both sexes (*p* < .05). Back scratch showed significant age difference among women but not men. BMI had no significant age difference in both men and women.

In all AGs, women showed better performance in the chair sit-and-reach test than men (ES = .34–.68) (*p* < .05). In addition, women had better performance in the back scratch test at AG1–3 (ES = .68–.74) than men (*p* < .001). BMI was larger in men than women (*p* < .05) in all AGs except at AG2 (ES = .36–.46).

Men had the highest decline rate in the chair sit-and-reach test (483.3%), while women had the highest decline rate in the back scratch test (236.0%) and the chair sit-and-reach test (114.2%). The lowest decline rate was found in the BMI (men: 2.0%; women: 3.1%).

### 3.3 Age- and sex-specific normative-referenced percentile values for seven indices of the SFT

**FIGURE 2:**
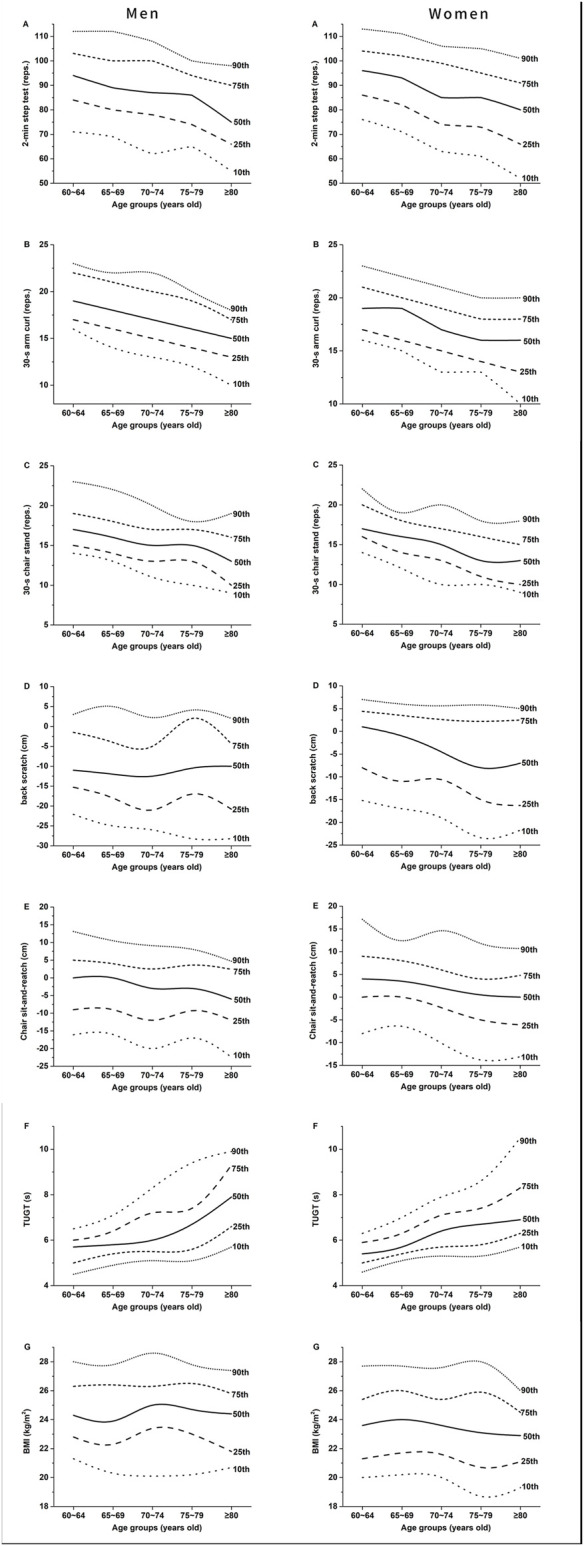
Age-specific normative values for each index of SFT in men [**(A–G)** in the left] and women [**(A–G)** in the right].

The normative-referenced percentile values at the 5th, 10th, 25th, 35th, 50th, 65th, 75th, 90th, and 95th percentiles for each index of SFT among the different AGs for men and women are listed in Tables 4, 5. Percentile-based changing trends with age for each index of SFT in men and women are shown in Figure 2 (for convenience, only the 10th, 25th, 50th, 75th, and 90th percentiles values are included). For easy comparison, norm values in the available literature and in the present study are integrated in Supplementary Table S2.

**TABLE 4:** Percentile norms by age group in women.

	Percentiles								
	5th	10th	25th	35th	50th	65th	75th	90th	95th
2-min step test (reps.)									
AG1	70	76	86	90	96	100	104	113	119
AG2	63	71	82	87	93	97	102	111	116
AG3	50	63	74	79	85	93	99	106	114
AG4	44	61	73	78	85	92	95	105	110
AG5	42	52	66	69	80	87	91	101	108
30-s arm curl (reps.)									
AG1	15	16	17	18	19	20	21	23	25
AG2	13	15	16	17	19	20	20	22	23
AG3	12	13	15	16	17	18	19	21	23
AG4	11	13	14	15	16	18	18	20	21
AG5	8	10	13	14	16	17	18	20	21
30-s chair stand (reps.)									
AG1	13	14	16	16	17	19	20	22	24
AG2	11	12	14	15	16	17	18	19	20
AG3	9	10	13	13	15	16	17	20	22
AG4	9	10	11	12	13	15	16	18	19
AG5	7	9	10	12	13	14	15	18	20
Back scratch (cm)									
AG1	−20.5	−15.2	−8.0	−5.0	1.0	2.9	4.4	7.0	8.6
AG2	−22.2	−17.0	−11.0	−7.2	−1.0	2.0	3.5	6.0	8.0
AG3	−23.1	−19.0	−10.6	−8.0	−4.5	2.0	2.6	5.6	7.5
AG4	−25.0	−23.4	−15.0	−12.0	−8.0	−2.7	2.2	5.8	8.8
AG5	−26.7	−21.7	−16.3	−10.0	−7.0	−2.6	2.5	5.0	5.0
Chair sit-and-reach (cm)									
AG1	−12.7	−8.0	.0	1.6	4.0	7.0	9.0	17.1	19.0
AG2	−13.0	−6.4	.0	2.0	3.5	5.5	8.0	12.4	16.2
AG3	−16.0	−10.1	−2.3	.0	2.0	4.0	6.0	14.6	20.0
AG4	−19.6	−13.8	−5.0	.0	.5	2.7	4.0	11.8	15.6
AG5	−20.0	−13.1	−6.1	−1.9	.0	2.0	4.8	10.7	14.4
TUGT (s)									
AG1	6.6	6.3	5.9	5.7	5.4	5.2	5.0	4.6	4.5
AG2	7.8	7.0	6.3	6.0	5.7	5.5	5.4	5.1	4.7
AG3	8.1	7.9	7.1	6.8	6.4	6.0	5.7	5.3	5.0
AG4	9.4	8.6	7.4	7.2	6.7	6.1	5.8	5.3	4.9
AG5	11.5	10.5	8.3	7.6	6.9	6.6	6.3	5.7	5.3
BMI (kg/m^2^)									
AG1	19.2	20.0	21.3	22.4	23.6	24.5	25.4	27.7	29.0
AG2	19.4	20.2	21.7	22.6	24.0	25.0	26.0	27.7	28.8
AG3	18.5	20.0	21.6	22.6	23.6	24.8	25.4	27.6	28.3
AG4	17.8	18.7	20.7	21.7	23.1	24.7	25.9	28.0	29.6
AG5	17.0	19.3	21.1	22.1	22.9	24.1	24.5	26.0	26.7

AG1, 60–64 years old; AG2, 65–69 years old; AG3, 70–74 years old; AG4, 75–79 years old; and AG5, ≥80 years old.

**TABLE 5:** Percentile norms by age group in men.

	Percentiles								
	5th	10th	25th	35th	50th	65th	75th	90th	95th
2-min step test (reps.)									
AG1	64	71	84	88	94	100	103	112	120
AG2	60	69	80	83	89	94	100	112	118
AG3	55	62	78	82	87	92	100	108	113
AG4	58	65	74	79	86	91	94	100	106
AG5	49	55	66	68	75	82	90	98	107
30-s arm curl (reps.)									
AG1	15	16	17	18	19	21	22	23	26
AG2	12	14	16	17	18	20	21	22	24
AG3	11	13	15	16	17	18	20	22	23
AG4	11	12	14	15	16	17	19	20	21
AG5	10	10	13	14	15	16	17	18	20
30-s chair stand (reps.)									
AG1	13	14	15	16	17	18	19	23	24
AG2	11	13	14	15	16	17	18	22	23
AG3	9	11	13	14	15	16	17	20	22
AG4	8	10	13	14	15	16	17	18	20
AG5	7	9	10	12	13	15	16	19	20
Back scratch (cm)									
AG1	−25.1	−22.1	−15.3	−13.0	−11.0	−5.7	−1.5	3.0	6.0
AG2	−31.0	−25.0	−18.0	−15.0	−12.0	−8.0	−4.0	5.0	7.0
AG3	−32.5	−26.0	−21.0	−18.4	−12.5	−8.0	−5.0	2.2	8.1
AG4	−34.0	−28.2	−17.0	−14.7	−10.5	−6.0	2.0	4.1	6.1
AG5	−40.0	−28.2	−20.8	−14.9	−10.0	−8.0	−4.4	2.0	7.6
Chair sit-and-reach (cm)									
AG1	−20.0	−16.1	−9.0	−5.0	.0	3.0	5.0	13.1	18.0
AG2	−20.0	−16.0	−9.0	−6.0	.0	2.5	4.0	10.5	16.0
AG3	−27.2	−20.0	−12.0	−7.0	−3.0	.0	2.5	9.1	18.4
AG4	−19.0	−17.0	−9.3	−8.2	−3.0	.0	3.6	8.1	13.6
AG5	−29.9	−22.4	−12.0	−10.0	−6.0	.0	2.4	4.7	9.3
TUGT (s)									
AG1	6.9	6.5	6.0	5.9	5.7	5.4	5.0	4.5	3.9
AG2	7.8	7.1	6.4	6.2	5.8	5.5	5.4	4.9	4.5
AG3	9.0	8.3	7.2	6.7	6.0	5.7	5.5	5.1	4.4
AG4	9.9	9.4	7.4	7.1	6.7	6.0	5.6	5.1	4.6
AG5	12.3	9.9	9.3	8.8	7.9	7.2	6.6	5.7	5.0
BMI (kg/m^2^)									
AG1	19.5	21.3	22.8	23.5	24.3	25.6	26.3	28.0	29.0
AG2	19.2	20.3	22.3	23.0	23.9	25.4	26.4	27.8	29.3
AG3	19.3	20.1	23.4	24.1	25.0	25.9	26.3	28.6	29.8
AG4	19.0	20.2	23.0	23.8	24.7	25.8	26.5	27.8	28.7
AG5	19.8	20.7	21.8	23.0	24.4	25.2	25.8	27.4	29.0

AG1, 60–64 years old; AG2, 65–69 years old; AG3, 70–74 years old; AG4, 75–79 years old; and AG5, ≥80 years old.

### 3.4 Computation of the SFT integrated score

To compute the SFT integrated score, the percentile norms among the participants of all ages and both sexes in the construction group were first analyzed (Table 6). Based on the results, the percentile method was applied to construct scores for each index (Table 7) with tipping points of 10th, 35th, 65th and 90th (Table 6). Thereafter, the weight of each SFT index score was obtained through factor analysis. The results of Bartlett sphericity test (*χ* ^*2*^ = 1,408.118, *p* < .001) and Kaiser–Meyer–Olkin (value = .816, > .8) demonstrated the suitability for factor analysis. Through principal component method in factor analysis, the component score coefficient matrixes of two common factors (F_1_ and F_2_) were extracted (Table 8):

$$F_{1}=0.298X_{1}+0.310X_{2}+0.344X_{3}—0.051X_{4}—0.014X_{5}+0.311X_{6}—0.145X_{7}$$

$$F_{2}=—0.059X_{1}—0.082X_{2}—0.139X_{3}+0.609X_{4}+0.563X_{5}—0.048X_{6}+0.396X_{7}$$

**TABLE 6:** The percentile norms deduced from all older adults in construction group.

Items	Percentiles								
	5th	10th	25th	35th	50th	65th	75th	90th	95th
2-min step test (reps.)	59	66	78	83	89	95	100	109	115
30-s arm curl (reps.)	13	14	16	17	18	19	20	22	24
30-s chair stand (reps.)	10	11	13	14	16	17	18	20	23
Back scratch (cm)	−26.0	−22.0	−15.0	−11.5	−7.0	.0	2.0	6.0	7.0
Chair sit-and-reach (cm)	−17.9	−14.0	−6.0	.0	1.0	4.0	6.0	12.7	17.0
TUGT (s)	8.7	7.8	6.8	6.3	5.9	5.6	5.4	4.9	4.5
BMI (kg/m^2^)	19.2	20.0	21.7	22.7	24.0	25.1	26.0	27.7	28.8

**TABLE 7:** Score of each SFT index deduced from all older adults in the construction group.

Items	Grade scores				
	1	2	3	4	5
2-min step test (reps.)	<66	66–82	83–94	95–108	≥109
30-s arm curl (reps.)	<14	14–16	17–18	19–21	≥22
30-s chair stand (reps.)	<11	11–13	14–16	17–19	≥20
Back scratch (cm)	<−22	−22.0∼−11.6	−11.5∼−.1	.0–5.9	≥6.0
Chair sit-and-reach (cm)	<−14	−14.0∼−.1	.0–3.9	4.0–12.6	≥12.7
TUGT(s)	≥7.8	6.3–7.7	5.6–6.2	4.9–5.5	<4.9
BMI(kg/m^2^)	<20.00,≥27.68		20.00–22.65,25.11–27.67		22.66–25.10

**TABLE 8:** Component score coefficient matrix of the two common factors (F_1_ and F_2_) within SFT exacted by the principal component method in factor analysis.

Items	Component	
	1	2
(X_1_) 2-min step test score	.298	−.059
(X_2_) 30-s arm curl score	.310	−.082
(X_3_) 30-s chair stand score	.334	−.139
(X_4_) Back scratch score	−.051	.609
(X_5_) Chair sit-and-reach score	−.014	.563
(X_6_) TUGT score	.311	−.048
(X_7_) BMI score	−.145	.396

The SFT integrated score was calculated with the variance contribution rate of the two common factors as the weight: F = Σ[λn/(λ1 + λ2)*Fn] (In this study, the cumulative variance contribution rate was 56.81%; F_1_ variance contribution rate was 41.8%; and the F_2_ variance contribution rate was 15.02%). The SFT integrated score formula was F = 73.6%F1 + 26.4%F2. Then, the actual weight of each index in the original formula was determined by the reference range of each index coefficient and the score in the comprehensive factor score formula, that is, the actual weight of Xi = 20 × Ci/Σ(C1 + Cn) (Tang et al., 2020). Finally, the SFT integrated score was obtained as 3.8 × 2-min step test score + 3.8 × 30-s arm curl score +3.8 × 30-s chair stand score + 2.2 × back starch score + 2.6 × chair sit-and-reach score +4 × TUGT score − .04 × BMI score. The SFT integrated score was calculated by substituting the SFT single index scores (Table 7) into the formula.

### 3.5 Verification of the integrated score

The SFT integrated score was verified using the error rate. The SFT integrated scores of the construction and validation groups were divided into five grades with an assigned score of 1–5, and the percentage of participants of each score was calculated (Table 9). The SFT integrated score of the construction group was set as the theoretical percentage, while that of the verification group was set as the actual percentage. The error rate was calculated as follows: (actual percentage − theoretical percentage)/theoretical percentage. The results showed that the error rates in each grade score were less than 5% (Table 9).

**TABLE 9:** Case and percentage under each SFT integrated grade score and the error rate between the construction and verification groups.

Group	Grade scores				
	1	2	3	4	5
Construction [*n* (%)]	78 (9.9)	193 (24.6)	233 (29.7)	201 (25.6)	80 (10.2)
Verification [*n* (%)]	35 (10.4)	81 (24.0)	99 (29.4)	88 (26.1)	34 (10.1)
Error rate (%)	5	−2.4	−1	2	−1

## 4 Discussion

In this study, SFT was measured in 1,122 community-dwelling older adults in Suzhou, China. The normative values of functional fitness in the different age stages in both men and women were established. More importantly, the SFT integrated score was established and verified based on the data from the randomly divided construction and verification groups.

As an internationally recognized physical fitness test tool for the older adults, the SFT has a comprehensive set of indices and is simple, practical for a wide range of people. More than 97% of the participants in our study completed the tests without adverse events, indicating the high safety, feasibility, and acceptance of the SFT in the old population. The adoption of SFT measurement enables the international comparison among different populations. Few studies had investigated the normative values of functional fitness levels in Chinese (Chen et al., 2009; Wang, 2015; Chung et al., 2016; Lin, 2020; Zhao et al., 2021). However, the SFT integrated score is missing in nearly all published studies (Chen et al., 2009; Wang, 2015; Chung et al., 2016; Zhao et al., 2021), and some of these reports used different parameters with the SFT (Chen et al., 2009; Wang, 2015; Lin, 2020). In the present study, normative values of functional fitness were established according to sex and age by using the data of SFT in 1,122 older adults in Suzhou, China. The results added new data to the current research and provided reference for the construction of national SFT normative values. Meanwhile, the SFT integrated score formula was provided to find a more concise way to evaluate the overall physical function.

To elucidate the difference between our normative values and previously published ones, the available percentile values at 50th are listed in Supplementary Table S2. References are marked in the Supplementary Table S2 and, for simplicity, are not marked in the subsequent comparisons in this paragraph. First, our participants had better aerobic capacity (2-min step test) results than those in India and Anhui of China but similar to those in the United States, Nanjing, HK, and Taiwan. Surprisingly, the results at Lanzhou for China were extremely higher, that is, approximately twice our values. We infer that the discrepancy was due to the failure in the explanation of the operation procedure in their research (which is referred in their article). Among the Chinese populations, the one in Anhui had lowest aerobic capacity, which may be related to its lower economic level and the resulting difference in health status and healthcare quality. Second, for the upper limb muscle strength (30-s arm-curl test), our participants had better results than those from India, Anhui, and HK but similar to all others, except for the larger results in Chilean women (male Chilean results are missing). Our results for the lower limb muscle strength (30-s chair stand test) were similar to previously published data with the lowest data from Indian and Anhui of China. Third, for the upper body flexibility (back scratch), most of our results were worse than those from the United States, better than those in the Portuguese, Spanish, Indian, and the Chinese of Lanzhou and Anhui, and close to the studies in Poland, Chile (only women available), Nanjing, and HK. For the lower body flexibility (chair sit-and-reach), our results were slightly worse than those from the United States, similar to those from Spain, Lanzhou, Taiwan, HK, Nanjing, and Poland, and better than those from Portugal, India, and Anhui of China. Fourth, our results in the agility and dynamic balance (TUGT) were similar to most of other results with slightly worse performance in Spain, India, and Anhui of China, and the older adults aged over 80 years old in Portugal. Lastly, our BMIs were smaller than those in the United States, Portugal, and Spain but similar to those in India, Nanjing, HK, and Taiwan. Therefore, most aspects of the SFT in our participants were better than those in India and Anhui of China but similar to other countries or regions with the exceptions in the lower body flexibility in the United States and the BMI.

The functional fitness level decreased with age. However, the decline rates in the different dimensions varied. The results showed that the flexibility of the upper and lower body declined most rapidly in women (236.0% and 114.2%) and upper body flexibility in men (483.3%), which were consistent with previous results in Nanjing (Zhao et al., 2021), HK (Chung et al., 2016), Portugal (Marques et al., 2014), and Poland (Ignasiak et al., 2020). Contrary to the information provided in Table 5, the report on Poland erroneously stated the following: The largest deficits involved the dynamic balance, with the reduction rate reaching 69% in men and 62% in women. Flexibility reflects the range of motion of each joint of the human body. The wide range of shoulder abduction and hip flexion may be associated with a large potential for change (Stathokostas et al., 2013). The largest reduction in flexibility may also related to the lack of flexibility exercise in older adults.

In addition to the greatest reduction in flexibility, the agility and dynamic balance ability (tested by TUGT) decreased continuously from AG1 to AG5 in men and women, and the same decline was found in other studies (Rikli and Jonesx, 1999b; Marques et al., 2014; Li et al., 2022). The most rapid decline in the dynamic balance ability was found in men after 80 years old (ES = .76). Hence, more attention should be paid to prevent the decline in the elderly at this age. In addition, the decline rates in the upper and lower limb muscle strength in men (21.3% and 23.1%) and women (20.3% and 24.7%) were similar to the results of men in HK (23.8% and 22.5%), but the decline rates in the HK elderly women (6.6% and 14.3%) were considerably smaller (Chung et al., 2016). Whether this result was related to different exercise levels or styles needs to be proved by further study. The decline rate of the lower limb muscle strength was slightly larger than that of the upper limb, which was also consistent in the United States (Rikli and Jones, 1999b) and Portugal (Marques et al., 2014), suggesting that more attention should be paid to lower limb muscle strength training. Similar to the study in Nanjing (Zhao et al., 2021), no significant age-related changes in the BMI were observed in this study. However, this result does not deny the potential link between BMI and age found in previous studies. After 60 years old, the average body weight tends to decrease (Seidell and Visscher, 2000), muscle will be gradually replaced by fat with an increase in age (Stathokostas et al., 2013), while independent activity ability will gradually decline.

Comparison of the sex differences of the various functional fitness indices showed that the women’s flexibility was better than men’s (ES: .24–.74). The same results were found in the United States (Rikli and Jones, 1999b) and Portugal (Marques et al., 2014). This result may be attributed to differences in tissue structure and bone morphology between men and women (Holland et al., 2002).

Functional fitness contains several aspects of physical capacities, which can be viewed either as a comprehensive physical quality or as different specific aspects of the physical capacities. The SFT is a battery test covering seven indices and five dimensions of physical fitness. In addition to the evaluation criteria of each index of the SFT, having an integrated score is necessary to show the overall physical capacity. A simple example of this need is the grade point average (GPA), which is calculated based on the scores of different courses and used to measure a student’s overall grade performance on average. An SFT integrated score also makes it convenient to set a single primary outcome in research. For example, in a study in which a comprehensive intervention of functional fitness is applied, the integrated score of SFT, instead of the scores of each index of SFT, may be desired as the primary outcome.

Previous studies have applied different methods to yield a comprehensive evaluation criteria of physical fitness. First, some researchers just simply combined the scores of all indices of SFT to yield the SFT integrated score (Wu and Zhao, 2021). Nevertheless, this computing method is simple and lacks accuracy. Due to the different units of the indices within the SFT, adding all values is unreasonable. Second, some studies used Z score to standardize the result of each index within the SFT [Z= (observed value − sample mean)/sample standard deviation] and then added the z-scores of each index together (Sardinha et al., 2015). Third, numerical normalization method was applied to obtain the normalized scores of each index and the normalized scores were added together (Jantunen et al., 2017). Lin (2020) constructed the comprehensive evaluation criteria for various dimensions of health-related functional fitness (ten tests were involved, and some duplicated the tests in the SFT) based on rank-sum ratio method. All these computing methods failed to consider the weight of each dimension of physical fitness, which may result in bias for the integrated score.

Factor analysis was used in this study. This process ensured that the weight proportion of the seven indices of the functional fitness could be objectively and scientifically assigned within the SFT integrated score. Therefore, the accuracy was improved. Two main common factors were extracted by factor analysis, and the weights were initially assigned to 73.6% and 26.4%. Then, the formula of the SFT integrated score was finally obtained after index coefficient correction. Xu (2015) studied older adults by carrying out factor analysis by using 15 fitness indices, and four common factors were extracted with assigned weights. However, the weight of each index was not integrated into the formulae. Meanwhile, the test contained quite many indices, thereby the process was time-consuming. In the integrated score formula in our study, coefficients of muscle strength, cardiopulmonary endurance, and dynamic balance index were larger. This result suggested that these three dimensions play important roles in the functional fitness level in older adults. These findings were consistent with our experience that cardiopulmonary endurance and muscle strength constitute the two core dimensions of physical fitness, and the dynamic balance ability is a comprehensive index involving muscle strength, nervous system flexibility, balance ability, and many other qualities. Thus, more attention should be paid to cardiopulmonary endurance, muscle strength, and dynamic balance ability during the evaluation and training of physical fitness among older adults.

The SFT integrated score was verified by calculating the error rates between the construction and verification groups. This method was applied in a previous study to examine the effectiveness of the reference standard of BMI percentile curve for children aged 3–6 years in China (Tu et al., 2021). In this method, the small error rate indicates the good consistency between the data for construction and for verification and the high reliability of the established formula or score (Wen, 2007). The error rates lower than 5% between the two groups in our study indicated the reasonability of the constructed SFT integrated score.

The establishment of the SFT normative values provides reference for older adults and healthcare workers to easily understand the measured levels and simplifies the comparison among the older adults and among the populations of different regions and ethnicities. However, several limitations exist. First, our participants were limited to the community-dwelling older adults in Suzhou, China, and the applicability of the results in other regions or in hospitalized patients needs to be further verified. In the future, large-scale and multicenter studies are required to obtain national results. Second, the SFT integrated score was constructed based on the ranked (1–5) instead of continuous scores of each index within the SFT. This process may reduce the assessment sensitivity. Future study can explore the method to construct the SFT integrated score based on continuous scores of each index. Third, due to the COVID-19 epidemic, the participant recruitment were frequently interrupted and suffered a lot of difficulties. Although it was planned that the participants be recruited according to the real proportion of the sex and age distribution of the older adults in Suzhou, the actually recruited samples were not exactly consistent with the real proportion. Compared with the population in the city, more women and less elderly people over 80 years were contained in our study, which may cause deviation in the integrated SFT score. However, since the normative values of each SFT index was built in age and sex specific older adults, they would not be affected. In future study, pay more attention should be paid to it and improve the deficiency.

## 5 Conclusion

In this study, based on the community-dwelling older adults in Suzhou, China, the functional fitness normative values for each index of the SFT and the SFT integrated score formula (SFT integrated score = 3.8 × 2-min step test score + 3.8 × 30-s arm curl score + 3.8 × 30-s chair stand score + 2.2 × back starch score + 2.6 × chair sit-and-reach score + 4 × TUGT score − .04 × BMI score) were established. The SFT integrated score formula was verified to be reasonable and effective.

## Data Availability

The raw data supporting the conclusion of this article will be made available by the authors, without undue reservation.
