# Supplementary material for: Normative values and integrated score of functional fitness among Chinese community-dwelling older adults in Suzhou
Source: Front Physiol. 2022 Dec 19;13:1063888. doi: 10.3389/fphys.2022.1063888 (PMC9806264; doi:10.3389/fphys.2022.1063888)
Supplement: Supplementary file 1 [file Table1.pdf]

S-Table 1 The proportion of male and female older adults in different age groups in our study and in Suzhou city (%).

Older adults	Sex	Age groups					Total
		60-64	65-69	70-74	75-79	≥80	
In our study	Male	10.5	10.6	8.5	7.0	4.3	40.8
	Female	16.8	19.2	12.3	7.2	3.7	59.2
	Total	27.3	29.8	20.8	14.2	8.0	100.0
In Suzhou city*	Male	13.3	13.8	9.3	5.6	5.8	47.8
	Female	13.4	14.2	9.8	6.2	8.5	52.2
	Total	26.7	28.0	19.2	11.8	14.3	100.0

*: obtained and calculated from the data of the seventh population census in 2020 in the webpage of https://www.hongheiku.com/shijirenkou/557.html

S-Table 2 Normative values at percentile of 50th for each index of SFT in men and women at different age groups in previous literature and our study.

P50	US (Rikli and Jones, 1999)	Spain (Gusi et al., 2012)	Portugal (Marques et al., 2014)	Chile (Valdés-Badilla et al., 2018)	Poland (Ignasiak et al., 2020)	India (Bhattacharya et al., 2017)	Taiwan (Chen et al., 2009)	HK (Chung et al., 2016)	Lanzhou (Lin, 2020)	Anhui (Wang, 2015)	Nanjing (Zhao et al., 2021)	Suzhou (the present study)
**2 min step test in men (reps.)**												
60~64	101	/	/	/	/	/	100.5	/	197	/	96	94
65~69	101					67	97	93	177	/	98	89
70~74	95					54	91.5	90	171	71	93	87
75~79	91					53.5	92	84	168	68	89	86
80~84	87					50[b]	80.5[b]	76	133[b]	64	78	75[b]
≥85	75/69[a]							/		56	77	
**2 min step test in women reps.)**												
60~64	91	/	/	114	/	/	92.5	/	187	/	106	96
65~69	90			108		64	92.5	82	186	/	96	93
70~74	84			100.5		52	90	84	175	61	94	85
75~79	84			101.5		37	90	81	147	59	85	85
80~84	75			96.5[b]		/	73[b]	74	118[b]	55	78	80[b]
≥85	70/58[a]					/		/		53	65	
**30-s arm curl in men (reps.)**												
60~64	19	/	/	/	20	/	/	/	24	/	22.5	19
65~69	18		19		20	11		14	21	/	18	18
70~74	17		18		18	11		12	21	17	17	17
75~79	16		16		18	9		12	19	15	15	16
80~84	16		14		16	11[b]		12	18[b]	12	13	15[b]
≥85	14/12[a]		13		14			/		8	13	

**30-s arm curl in women (reps.)**												
60~64	16	/	/	24	18	/	/	/	19	/	20	19
65~69	15		18	24	18	14		12	18	/	17	19
70~74	15		17	23	17	12.5		13	18	13	16	17
75~79	14		15	22	15	7		13	18	13	15	16
80~84	13		12	21[b]	15	/		12	16[b]	10	14	16[b]
≥85	12/11[a]		11		12	/		/		7	13	
**30-s chair stand in men (reps.)**												
60~64	16	/	/	/	16	/	17	/	16	/	17	17
65~69	15		16		16	15	15	17	14	/	16	16
70~74	15		15		15	13	14	15	14	14	15	15
75~79	14		13		14	9	12	14	14	12	14	15
80~84	12		12		13	10[b]	11[b]	12.5	11[b]	10	12	13[b]
≥85	11/10[a]		11		11			/		6	12	
**30-s chair stand in women (reps.)**												
60~64	15	/	/	17	15	/	14	/	14	/	17	17
65~69	14	/	15	17	15	12.5	13	15	13	/	16	16
70~74	13	/	15	16	14	9.5	12	15.5	14	11	15	15
75~79	12	/	13	16	13	5	12	14	12	10	14	13
80~84	11	/	10	16[b]	13	/	9.5[b]	13	11[b]	9	13	13[b]
≥85	10/8[a]	/	9		12	/		/		7	12	
**Back scratch in men (cm)**												
60~64	-3.5	-13	/	/	-8	/	/	/	-13.9	/	-12	-11
65~69	-4	-16	-15		-10	-6		-1	-16	/	-10	-12
70~74	-4.5	-17	-17		-10	-10		-8	-19	-12.5	-11	-12.5
75~79	-5.5	-19	-20		-13	-8.5		-12	-20.3	-17.5	-15	-10.5

80~84	-5.5	-23.5	-25		-14.5	-18[b]		-12.5	-21.4[b]	-25.5	-14	-10[b]
≥85	-6/-7[a]	-28	-28		-20			/		-32.1	-25	
**Back scratch in women (cm)**												
60~64	-0.5	-7	/	-5	0	/	/	/	-9.7	/	0.5	1
65~69	-1	-9	-10	-5.3	-1	-10		0.25	-10	/	-4	-1
70~74	-1.5	-10	-11	-8	-2	-12		1	-11	-15.1	-7	-4.5
75~79	-2	-12	-15.3	-9.9	-4	-34		-0.5	-11	-17.1	-9	-8
80~84	-2.5	-16	-21	-10.1[b]	-4	/		-2.5	-15[b]	-24.1	-9	-7[b]
≥85	-4.0/-4.5[a]	-17	-23		-10	/		/		-29.5	-11	
**Chair sit-and-reach in men (cm)**												
60~64	0.5	0	/	/	2	/	6.5	/	0	/	1	0
65~69	0	0	-6		0.5	-13	2	-1	-2	/	1	0
70~74	-0.5	-1	-8.5		0	-12	0	0	-3	-11.1	-1	-3
75~79	-1	-4	-9		1	-8	-3	-2	-5.9	-10.5	0	-3
80~84	-2	0	-14		0	-20[b]	-4[b]	-7.5	-7.4[b]	-16.5	-8	-6[b]
≥85	-2.5/-3.5[a]	-12	-15		-1.5			/		-21.5	-10.5	
**Chair sit-and-reach in women (cm)**												
60~64	2	2	/	5.7	4	/	6	/	3	/	6	4
65~69	2	1	0	3.4	3	-7	6	6	2.2	/	6	3.5
70~74	1.5	1	-1	3.5	2	-19.5	4	6.5	1	-3.5	4.8	2
75~79	1	0	-2	2.5	2	-24	3.8	4	0.5	-5.5	3	0.5
80~84	0.5	0	-10	1.2[b]	2	/	0[b]	1	-1.9[b]	-8.5	1	0[b]
≥85	-0.5/-2[a]	0	-13		0	/		/		-12.5	-2	
**TUGT in men (s)**												
60~64	4.7	7.1	/	/	5.3	/	/	/	6.3	/	5.4	5.7
65~69	5.1	7.1	5.1		5.5	7		4.84	7	/	5.3	5.8

70~74	5.3	7.3	5.9		5.8	7.2		5.58	7.6	7.4	5.9	6
75~79	5.9	7.8	6.9		6	8.3		6.1	7.7	8.2	6.3	6.7
80~84	6.4	9.75	8.3		7	13.2[b]		6.99	10[b]	9.3	7.3	7.9[b]
≥85	7.2/8.1[a]	10.2	10.1		8.2			/		10.6	8.8	
**TUGT in women (s)**												
60~64	5.2	7.2	/	5	5.7	/	/	/	6.8	/	5	5.4
65~69	5.6	7.6	5.6	5.2	5.9	7.7		5.97	7.1	/	5.6	5.7
70~74	6	8	6	5.4	6.3	10.7		5.86	8.1	9.6	5.9	6.4
75~79	6.3	8.63	7.3	5.8	7	20.3		6.66	8.4	10.2	6.8	6.7
80~84	7.2	10	10.6	6.2[b]	7.3	/		7.5	9.9[b]	11.8	7.9	6.9[b]
≥85	7.9/9.4[a]	12.43	12.6		8.2	/		/		13.6	9.2	
**BMI in men (kg/m^2)**												
60~64	27.4	30	/	/	/	/	24.4	/	/	/	25.1	24.3
65~69	27.5	29.4	27.6			23.63	24.5	23.6			24.8	23.9
70~74	26.6	29.3	27.4			23.18	24.5	24.6			25	25
75~79	26.4	29.5	27.2			22.35	23.4	24.5			24.8	24.7
80~84	26.1	28.5	27.1			21.54[b]	23[b]	23.4			24.2	24.4[b]
≥85	24.9/24.9[a]	28.2	26.4					/			24.3	
**BMI in women (kg/m^2)**												
60~64	26.3	30.7	/	/	/	/	23.3	/	/	/	24.2	23.6
65~69	26.5	30.7	28.2			20.31	24.6	24			24.7	24
70~74	26.1	30.5	27.9			23.23	24.3	23.6			24.5	23.6
75~79	25.4	30.4	27.8			22.93	23.6	23.8			24.3	23.1
80~84	24.7	30.1	27.8			/	23.1[b]	23.6			23.5	22.9[b]
≥85	24.3/24.1[a]	28.7	27			/		/			24	

/: the values are not tested, [a]: the values of 85~89 (before/) and 90~94 (after/) years old respectively, [b]: the values for all older adults ≥80 years old, and they are same for all parameters.

References

Bhattacharya, P.K., Deka, K., Roy, A., Saikia, H.J.J.o.C., and Research, D. (2017). Normative Values of Physical Fitness Test in the Elderly: A Community Based Study in an Urban Population in Northeast India. 11(10).

Chen, H.-T., Lin, C.-H., and Yu, L.-H.J.J.o.N.R. (2009). Normative physical fitness scores for community-dwelling older adults. 17(1), 30-41.

Chung, P.K., Zhao, Y., Liu, J.D., and Quach, B. (2016). Functional fitness norms for community-dwelling older adults in Hong Kong. *Arch Gerontol Geriatr* 65, 54-62. doi: 10.1016/j.archger.2016.03.006.

Gusi, N., Prieto, J., Olivares, P.R., Delgado, S., Quesada, F., Cebrián, C.J.J.o.a., et al. (2012). Normative fitness performance scores of community-dwelling older adults in Spain. 20(1), 106-126.

Ignasiak, Z., Sebastjan, A., Sławińska, T., Skrzek, A., Czarny, W., Król, P., et al. (2020). Functional fitness normative values for elderly polish population. 20(1), 1-9.

Lin, D.Y. (2020). *A Study on the Evaluation Criteria of Healthy Physical Fitness for the Elderly in Lanz.* master, Normal University of Northwest.

Marques, E.A., Baptista, F., Santos, R., Vale, S., Santos, D.A., Silva, A.M., et al. (2014). Normative functional fitness standards and trends of Portuguese older adults: cross-cultural comparisons. 22(1), 126-137.

Rikli, R.E., and Jones, C.J. (1999). Functional fitness normative scores for community-residing older adults, ages 60-94. *Aging Phys Act* 7(2), 162-181.

Valdés-Badilla, P., Concha-Cisternas, Y., Guzmán-Muñoz, E., Ortega-Spuler, J., and Vargas-Vitoria, R. (2018). [Reference values for the senior fitness test in Chilean older women]. *Rev Med Chil* 146(10), 1143-1150. doi: 10.4067/s0034-98872018001001143.

Wang, H.Y. (2015). *The Establishment and Application of Health Fitness Evaluation Index System for Elderly People over 70 years.* Doctor, Soochow University.

Zhao, Y., Wang, Z., Chung, P.-K., and Wang, S.J.S.R. (2021). Functional fitness norms and trends of community-dwelling older adults in urban China. 11(1), 1-8.
